# Supplementary material for: Novel Driver Strength Index highlights important cancer genes in TCGA PanCanAtlas patients
Source: PeerJ. 2022 Aug 11;10:e13860. doi: 10.7717/peerj.13860 (PMC9375969; doi:10.7717/peerj.13860)
Supplement: Supplemental Information 2 [file peerj-10-13860-s002.zip › COHORTS/genes plots/age/2021_8_16_14_9_distribution_cohorts_35-39.pdf]

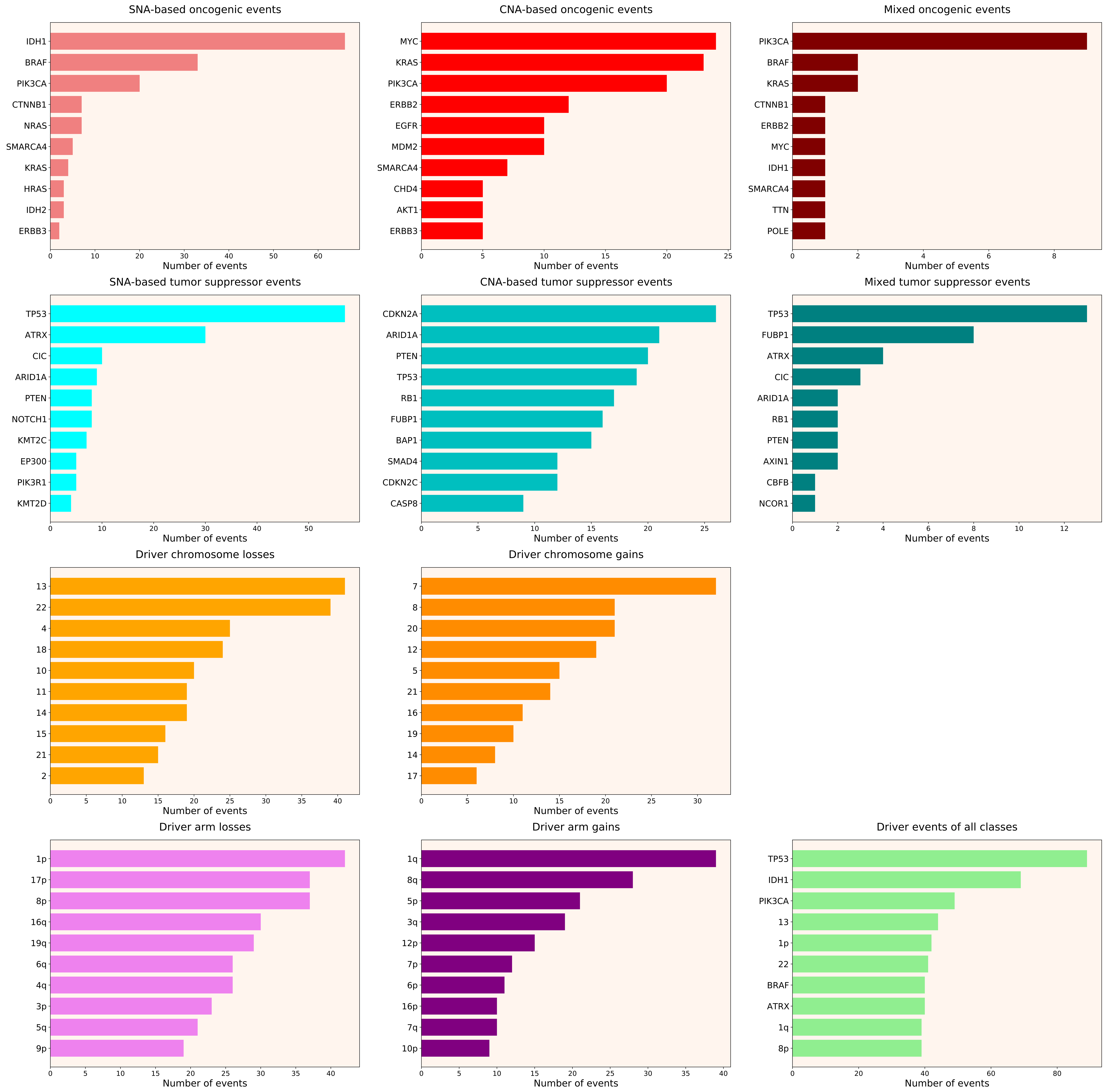
SNA-based oncogenic events
IDH1
BRAF
PIK3CA
CTNNB1
NRAS
SMARCA4
KRAS
HRAS
IDH2
ERBB3
0 10 20 30 40 50 60
Number of events
CNA-based oncogenic events
MYC
KRAS
PIK3CA
ERBB2
EGFR
MDM2
SMARCA4
CHD4
AKT1
ERBB3
0 5 10 15 20 25
Number of events
Mixed oncogenic events
PIK3CA
BRAF
KRAS
CTNNB1
ERBB2
MYC
IDH1
SMARCA4
TTN
POLE
0 2 4 6 8
Number of events
SNA-based tumor suppressor events
TP53
ATRX
CIC
ARID1A
PTEN
NOTCH1
KMT2C
EP300
PIK3R1
KMT2D
0 10 20 30 40 50
Number of events
CNA-based tumor suppressor events
CDKN2A
ARID1A
PTEN
TP53
RB1
FUBP1
BAP1
SMAD4
CDKN2C
CASP8
0 5 10 15 20 25
Number of events
Mixed tumor suppressor events
TP53
FUBP1
ATRX
CIC
ARID1A
RB1
PTEN
AXIN1
CBFB
NCOR1
0 2 4 6 8 10 12
Number of events
Driver chromosome losses
13
22
4
18
10
11
14
15
21
2
0 5 10 15 20 25 30 35 40
Number of events
Driver chromosome gains
7
8
20
12
5
21
16
19
14
17
0 5 10 15 20 25 30
Number of events
Driver arm losses
1p
17p
8p
16q
19q
6q
4q
3p
5q
9p
0 5 10 15 20 25 30 35 40
Number of events
Driver arm gains
1q
8q
5p
3q
12p
7p
6p
16p
7q
10p
0 5 10 15 20 25 30 35 40
Number of events
Driver events of all classes
TP53
IDH1
PIK3CA
13
1p
22
BRAF
ATRX
1q
8p
0 20 40 60 80
Number of events
